# Deep learning‐based synthetic‐CT‐free photon dose calculation in MR‐guided radiotherapy: A proof‐of‐concept study

**DOI:** 10.1002/mp.70106

**Published:** 2025-11-04

**Authors:** Fan Xiao, Domagoj Radonic, Niklas Wahl, Nikolaos Delopoulos, Adrian Thummerer, Stefanie Corradini, Claus Belka, George Dedes, Christopher Kurz, Guillaume Landry

**Affiliations:** ^1^ Department of Radiation Oncology LMU University Hospital LMU Munich Munich Germany; ^2^ Department of Medical Physics in Radiation Oncology German Cancer Research Center (DKFZ) Heidelberg Germany; ^3^ National Center for Radiation Oncology (NCRO) Heidelberg Institute for Radiation Oncology (HIRO) Heidelberg Germany; ^4^ German Cancer Consortium (DKTK) partner site Munich, a partnership between DKFZ and LMU University Hospital Munich Germany; ^5^ Bavarian Cancer Research Center (BZKF) Munich Germany; ^6^ Department of Medical Physics Faculty of Physics, Ludwig‐Maximilians‐Universität München Munich Germany

**Keywords:** deep learning, dose calculation, LSTM, MRI, online MR‐guided photon therapy

## Abstract

**Background:**

In magnetic resonance imaging (MRI)‐guided online adaptive radiotherapy, MRI lacks tissue attenuation information necessary for accurate dose calculations. Although deep learning (DL)‐based synthetic computed tomography (CT) generation models have been developed to obtain CT density information from MRI, they usually do not meet the requirement of real‐time plan adaptation.

**Purpose:**

We propose a DL‐based photon dose calculation method directly on 0.35 T MRI to skip synthetic CT generation and show its feasibility for prostate patient cases.

**Methods:**

The 0.35 T planning MRI and deformed planning CT (registered to the planning MRI) of 34 prostate cancer patients treated with a 0.35 T magnetic resonance‐linear accelerator (MR‐Linac) were collected. The air cavities (ACs) in the abdominopelvic area of the deformed CT images were corrected based on manual AC contouring on the MRI. Monte Carlo (MC) dose simulations under a 0.35 T magnetic field were performed on the corrected CT images. All photon beams were simulated using a uniform field size of 1cm×1cm. 10 800 beams were simulated with $5\times 10^{6}$ initial photons for training (20 patients) and 2160 beams with $5\times 10^{7}$ photons for validation (4 patients). For testing, 1080 beams shooting through the planning target volume (PTV) in 10 patients and five optimized nine‐field intensity‐modulated plans were simulated with $5\times 10^{7}$ photons. 3D MRI cuboids covering the photon beams were input into a Unet model to predict AC segmentation, and 3D MRI and predicted AC cuboids were input into a long short‐term memory (LSTM) model for beam's eye view (BEV) processing to predict dose. The gamma passing rate $\gamma_{\mathrm{pr}}$ (2%/2mm, $D>10\%D_{\max}$), beam dose profiles of single beams and dose volume histogram (DVH) of intensity‐modulated plans were evaluated.

**Results:**

The test results for all photon beams from the proposed models demonstrated a mean $\gamma_{\mathrm{pr}}$ above 99.50%. The five treatment plans recalculated by the DL model each achieved $\gamma_{\mathrm{pr}}$ values exceeding 99.80%. Additionally, the model's inference time was approximately 12 ms per photon beam.

**Conclusions:**

The proposed method showed that DL‐based dose calculation directly on MRI is feasible for prostate cases, which has the potential to simplify the procedure for MRI‐only workflows and can be beneficial for real‐time plan adaptation.

## 1 INTRODUCTION

Magnetic resonance imaging (MRI) is becoming widely used in radiotherapy because of its high soft tissue contrast to visualize target volumes and its image acquisition process based on nonionizing radiation.^1^ Magnetic resonance‐linear accelerators (MR‐Linacs)^2^, ^3^ combine a linac with an MR scanner, making MRI‐guided online adaptive radiotherapy feasible.^4^, ^5^ The daily treatment plan can be adapted to manage inter‐fractional variations, and intra‐fractional beam gating based on real‐time 2D cine MRI sequences^6^ can account for the target/tumor motion during treatment.^7^, ^8^ Ideally, the ultimate approach would be real‐time adaptation, which would rely on real‐time 3D MRI^9^, ^10^ to recalculate the delivered dose based on the latest geometry, and accumulate dose to re‐optimize the remaining dose within this fraction.^11^, ^12^

For plan re‐optimization, MRI cannot be used directly as it lacks electron density information required for accurate dose calculations. A variety of synthetic computed tomography (CT) generation methods from MRI have been developed^13^ and recently deep learning (DL)‐based methods showed promising results. Based on the SynthRAD2023 challenge report,^14^, ^15^ different DL models are proposed to generate 2D/3D synthetic CT from MRI and top‐performing teams achieved an average 99.0% gamma passing rates (2%/2 mm, 10% dose threshold of the prescribed dose) in photon treatment plans. However, the average inference time per patient for DL models was 5.2±2.8 min.^15^ Although DL‐based synthetic CT studies published in 2025^16^, ^17^ have reported average processing times of under 2 min per patient, these times remain acceptable for daily plan adaptation, but do not meet the requirement of real‐time adaptation.

On the other hand, fast dose calculations are critical for online plan re‐optimization and ultimately for accounting for intra‐fractional motion. For MR‐Linacs, the effect of the magnetic field on dose can be considered by Monte‐Carlo (MC) simulation.^18^ Although traditional MC methods^19^ are highly time‐consuming, graphics processing unit (GPU)‐based approaches^20^, ^21^ have accelerated MC photon dose calculation to a few or tens of seconds. Meanwhile, DL methods have achieved sub‐second^22^, ^23^ or nearly 1s^24^, ^25^ photon dose calculation speeds with promising accuracy, offering the possibility for real‐time plan adaptation.

Given that DL methods can accurately generate synthetic CT from MRI and perform dose calculations based on CT with high precision (to the best of our knowledge, all DL dose calculation methods are CT‐based), it would be highly valuable to explore the potential of DL methods for direct dose calculation on MRI, as also discussed in some studies.^26^, ^27^ By bypassing the time‐consuming synthetic CT generation process, DL dose caculation on MRI has the potential to support real‐time intra‐fractional plan adaptation based on 3D time‐resolved MRI from MR‐Linacs.^28^

In this work, building upon the previous long short‐term memory (LSTM)‐based proton dose calculation studies addressing dose calculation in beam's eye view (BEV),^29^, ^30^ we explore the use of Unet‐LSTM combined networks to directly build the mapping between the MRI and the photon beam dose simulated on the corresponding deformed CT. For this proof‐of‐concept experiment, we train, validate, and test the network on prostate cancer patient datasets, and all photon beams were simulated with a uniform small‐sized field.

## 2 MATERIALS AND METHODS

### 2.1 Patient data

The patient dataset consists of imaging data from 34 prostate cancer patients treated with the 0.35 T MR‐Linac (MRIdian, ViewRay, USA)^3^ at the Department of Radiation Oncology, LMU University Hospital. For each patient case, the planning MRIs were acquired at the 0.35 T MR‐Linac using a balanced steady state free‐precession (bSSFP) sequence (yielding a T2/T1‐weighted contrast).^31^ The planning CT images obtained from a single CT scanner (Aquilion LB, Canon Medical Systems, NL) were deformably registered to planning MRI in the ViewRay treatment planning system (TPS),^32^ using an intensity‐based algorithm. For each pair of planning MRI and deformed CT, the resulting registration was visually verified by physicians during the online adaptive workflow.^33^ The voxel size of both planning MRI and deformed planning CT was $1.5\times 1.5\times 1.5\;{\text{mm}}^{3}$. All patients were anonymized and had no artificial implants.

### 2.2 Air cavity (AC) correction

**FIGURE 1:**
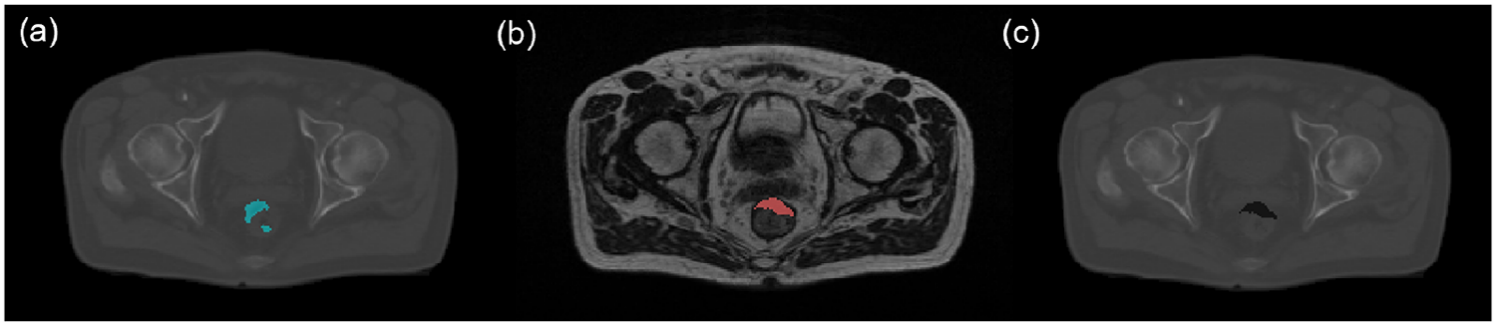
AC correction in the transverse plane of a prostate case: (a) deformed CT with filled AC (blue area), (b) AC contours on MRI (red area indicates the AC contour), (c) corrected CT. AC, air cavitie; CT, computed tomography; MRI, magnetic resonance imaging.

Although electron density transfer based on deformable image registration (DIR) can achieve high accuracy, it still has limitations when handling the addition or removal of new tissue compartments, especially in cases of air/gas volume changes within the abdominopelvic region.^34^ To take AC changes into account for accurate comparison of dose calculations on deformed CTs and MRIs, we applied AC correction to the deformed CT based on the corresponding MRI for each patient. As shown in Figure 1, the following three steps were performed: (1) Extract AC masks in deformed CT using Hounsfield Unit (HU) thresholding (CT values < ‐300 HU). For each unconnected component of the AC mask, calculate the median values of neighbor voxels and fill the corresponding AC region with these median values to obtain the filled CT. (2) Manually delineate AC contours on the MRI using a research version of a commercial TPS (RayStation, RaySearch Laboratories, Stockholm, Sweden), as long as the ACs were located within the peri‐prostatic region, regardless of their volume. Additionally, TotalSegmentator^35^ was applied to MRI to generate gas‐containing organ masks (colon, bowel, duodenum, rectum), enabling rough localization of gas‐containing organs to facilitate manual AC contouring. (3) Insert AC contours from MRI to filled CT and assign an AC value of −700 HU, as referenced in ref. [36]. We used the corrected CT for MC simulation to generate dose distributions for training and evaluating the model.

### 2.3 MC simulation

In this work, MC dose distributions were simulated using Geant4 v11.00‐patch‐03 with the predefined QGSP_BIC_HP_EMZ physics list. CT numbers of corrected CTs were converted to mass density and elemental composition using a scanner‐specific calibration curve established in a previous study.^37^ The photon energy spectrum we used in our Geant4 simulations was extracted from the ELEKTA_PRECISE_6MV phase space files provided and validated by the International Atomic Energy Agency (IAEA).^38^ A uniform 0.35 T magnetic field was simulated in Geant4, aligned parallel to the superior‐inferior patient direction and confined within a cylindrical region with a 50 cm diameter.^3^ The dose distribution was scored on the same voxel geometry as the corrected CT.

For this proof‐of‐concept study, all photon beams were simulated parallel to the transverse plane with a uniform square field size of $1\times 1\;{\text{cm}}^{2}$, based on a plane source with the extracted photon energy spectrum. The source‐to‐axis distance was set to 100 cm and gantry angles of photon beams were multiples of 10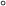. Twenty patients were randomly selected for training, 4 patients for validation and 10 patients for testing. For each training and validation patient, the isocenter was shifted to three positions along the anterior–posterior (AP) direction (0 cm, +3 cm, −3 cm) and five positions along the superior–inferior (SI) direction (−4 cm, −2 cm, 0 cm, +2 cm, +4 cm). For each isocenter position, 36 evenly spaced gantry angles (every 10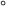 from 0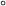 to 350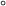) were simulated, resulting in a total of 36×3×5 = 540 beams per patient. For test patients, three random positions of iso center within or near the edge of the planning target volume (PTV) in superior‐inferior direction were selected, yielding 36×3 beams simulated per patient. In total, 10800 beams were simulated for 20 training patients, 2160 beams simulated for 4 validation patients, and 1080 beams through the PTV simulated for 10 test patients. Due to simulation time limitations (with a mean runtime of approximately 20 h for $5\times 10^{7}$ histories on a single core of an Intel Xeon Gold 6354 3.00 GHz CPU), each training beam was simulated with $5\times 10^{6}$ histories (with an average statistical uncertainty of approximately 4.5% in the $D>10\%D_{\max}$ region, and a runtime of 2 h), while each validation and test beam was simulated with $5\times 10^{7}$ histories (with an average statistical uncertainty of less than 1.5% in the $D>10\%D_{\max}$ region, and a runtime of 20 h).

In addition to the single beam evaluation, five patients were selected from the 10 test patients for evaluation of full treatment plans. Four cases were randomly selected (plans 1, 3, 4 and 5) and one case was selected with an AC near the PTV on the MRI (plan 2). Five nine‐field intensity‐modulated plans with gantry angles of 0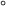, 40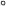, 80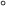, 120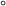, 160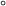, 200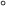, 240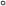, 280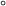, and 320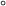 were generated and optimized with pyRadPlan v0.2.8,^39^ an open‐source Python treatment planning toolkit based on matRad.^40^, ^41^ Specifically, each field comprised 81 parallel beams with a uniform square field size of $1\times 1\;{\text{cm}}^{2}$, pre‐calulated using $5\times 10^{7}$ histories. The corrected CTs and PTV masks, along with the MC beam doses, were imported into pyRadPlan, where treatment plans were then optimized by weighting the individual $1\times 1\;{\text{cm}}^{2}$ beams using a quadratic difference objective for the PTV trading against a quadratic overdose objective for the normal tissue. The prescribed dose was 74Gy delivered in 37 fractions, which is a standard and commonly used conventional fractionation regimen for prostate cancer at regular linacs. Please keep in mind that the fractionation is not relevant to the relative dose accuracy evaluations done in this work. MC treatment plans were normalized such that $D_{95\%}$ of the PTV received at least 95% of the prescribed dose (i.e., 70.4Gy in this study). Then, the total MC and predicted doses of the five nine‐field plans, obtained using identical optimized weights and the same scaling factor applied for normalizing the MC plans, were compared (see section 2.6).

### 2.4 Pre‐processing

After the MC simulation, all values of the 3D photon beam dose and 3D MRI maps outside the patient's body were set to 0 based on the body contour from the 3D MRI. The intensity values of each 3D MRI volume were clipped at the 99.5th percentile to account for potential high‐intensity MRI artifacts. Patient‐specific maximum normalization was applied to the 3D MRI volumes, while global maximum normalization, using the maximum beam dose value from training beams, was applied to the 3D beam dose volumes of the training, validation, and testing datasets. Next, BEV cuboid extraction operations based on the beam direction and source position were applied on the normalized 3D beam dose and MRI volumes, as well as the 3D AC contour volumes. 3D cuboids of MRI, AC and dose maps were cropped from the patient's surface and resampled. The dimensions of all 3D cuboids (z×y×x) were set to 200×32×32, corresponding to the penetration depth z and the xy‐plane perpendicular to it (the x‐axis being parallel to the superior‐inferior direction), with a resolution of $2\times 2\times 2\;{\text{mm}}^{3}$. Besides, to consider the lateral dose shift and electron return effect (ERE) due to the magnetic field, the cuboid center was shifted 1 cm in the y‐axis with respect to the beam's center. To facilitate comparison with a CT‐based dose calculation model, the corresponding corrected CT cuboids using the same global normalization and BEV extraction operations were also collected.

### 2.5 Model architecture

**FIGURE 2:**
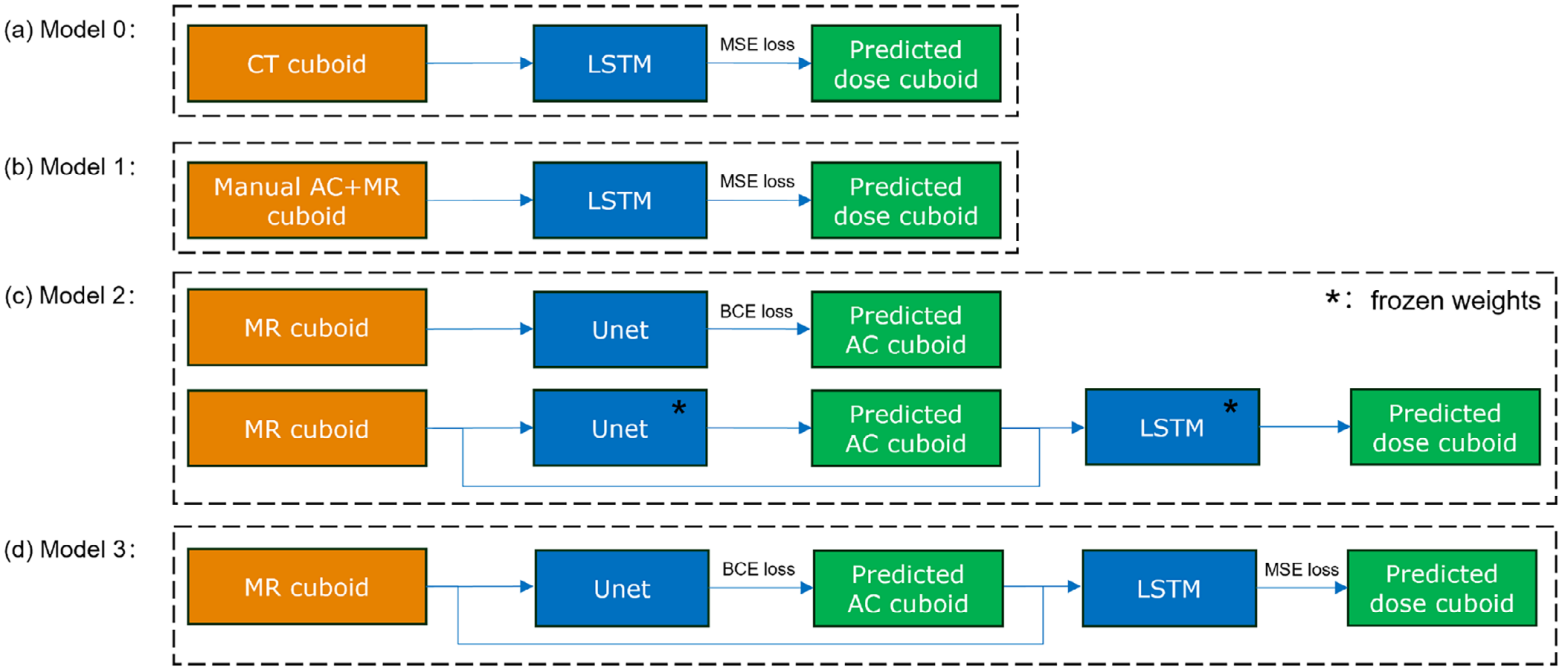
Overview of the four proposed models: (a) Model 0: the LSTM model converting CT to dose, (b) Model 1: the LSTM model converting MRI and manual AC to dose, (c) Model 2: the Unet model converting MRI to predicted AC, and the LSTM model from Model 1 converting MRI and predicted AC to dose, (d) Model 3: jointly trained Unet and LSTM models. Training was conducted using MSE loss for dose prediction and BCE loss for AC segmentation. Models with frozen weights in (c) refer to pre‐trained models for inference. AC, air cavities; BCE, binary cross‐entropy; CT, computed tomography; LSTM, long short‐term memory; MRI, magnetic resonance imaging; MSE, mean squared error.

Four models, as shown in Figure 2 were proposed: Model 0, serving as the baseline, was a CT‐based dose calculation model that took CT cuboids as inputs (with globally normalized HU values) and generated dose cuboids as outputs. The same LSTM model architecture in a previous study^30^ was used (no energy embedding layer), consisting of a one‐layer LSTM network and a two‐layer fully‐connected network, which converts a sequence of CT slices to a sequence of dose slices.

**FIGURE 3:**
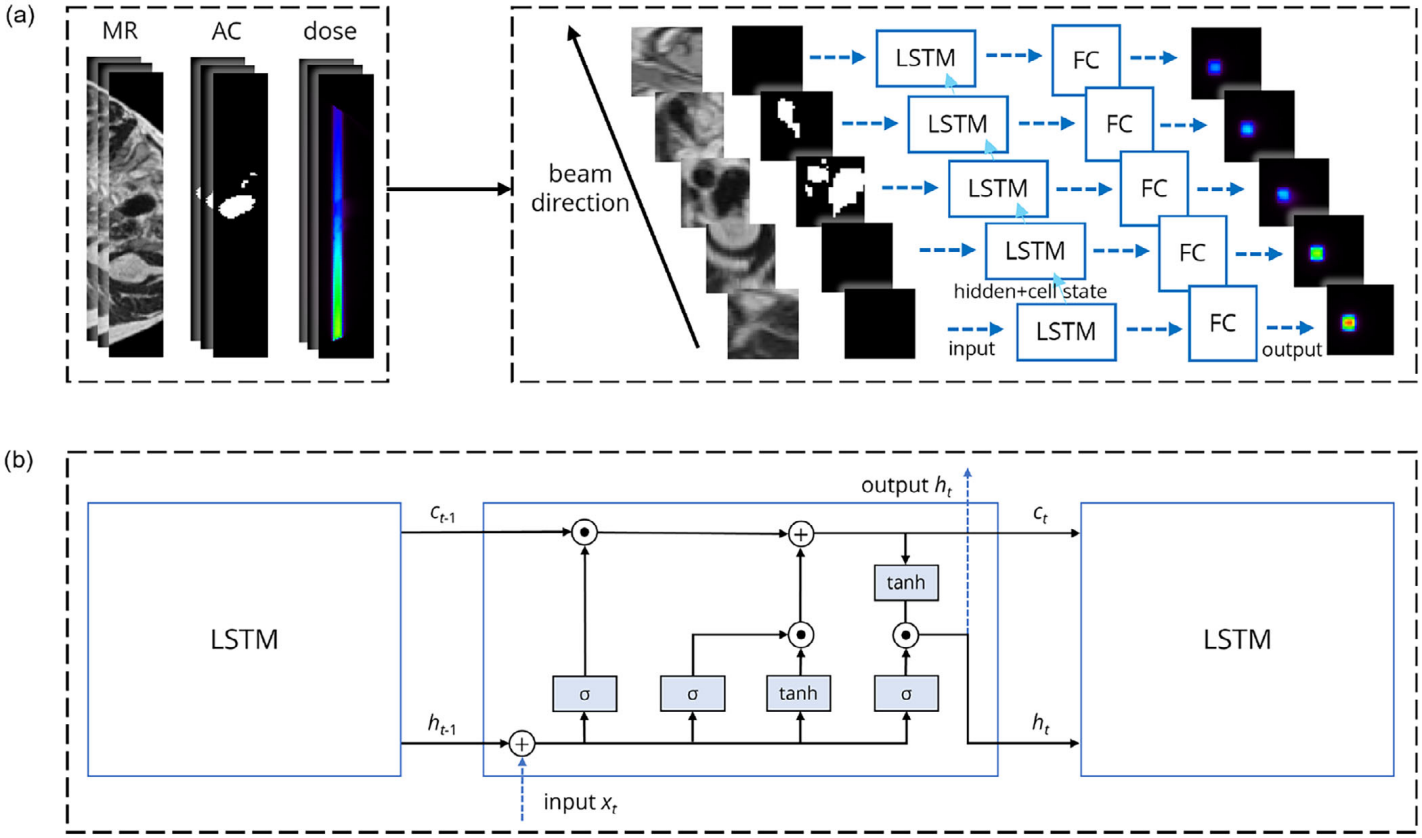
Training framework of Model 1: (a, left) The 3D input MRI and AC cuboid and ground truth dose cuboid after BEV resampling. (a, right) The slices of MRI and AC cuboid of the same depth in beam direction are concatenated and flattened into 1D input sequences, and the LSTM model, which consists of a one‐layer LSTM network and a two‐layer fully‐connected network (labeled as LSTM and FC), processes these combined sequences and outputs the predicted dose. (b) The corresponding LSTM module structure: $x_{t}$ is the 1D flattened input slice received at each step, while $h_{t}$ represents the corresponding 1D output sequence passed to the fully‐connected network. The cell states $c_{t-1}$ and $c_{t}$ (previous and current steps) and the hidden states $h_{t-1}$ and $h_{t}$ (previous and current steps) are for maintaining the LSTM's memory. The σ functions denote the input, forget, and output gates of the LSTM, whereas the tanh functions denote the updating of the cell state information. Adapted from ref. [42].

Unlike CT, which features distinguishable ACs with low‐intensity values, MRI presents numerous low‐signal areas that resemble ACs. This leads the LSTM Model to overlook the dose effects associated with ACs when relying solely on the MRI cuboid. To address this limitation, we introduce Model 1, which uses both MRI and manually contoured AC cuboids as inputs of the LSTM model to increase the model's focus to the AC area. It converts a sequence of MRI and AC slices to a sequence of dose slices (training framework shown in Figure 3, similar to a previous study^42^).

The manual AC contouring as an input for dose calculation is not realistic for online workflows. Thus, inspired by an AC segmentation study on MRI,^43^ we first trained an AC segmentation Unet model which can predict the 3D AC cuboid from the 3D MRI cuboid. Then, based on the trained LSTM model from Model 1 and the trained Unet, the dose cuboid can be inferred by inputting the MRI cuboid into the two models with frozen weights. As shown in Figure 2c, we refer to the models (LSTM and Unet) trained separately but used for joint inference as Model 2.

For Model 3, instead of training the Unet and LSTM models separately, we trained them jointly using a combined loss, which is the sum of the AC segmentation loss and the dose prediction loss. The models are also used jointly for inference, as shown in Figure 2d.

For the network architecture, the LSTM models used in the above four models are the same, and the Unet models used in Model 2 and Model 3 are the same. The LSTM model features one layer with a hidden state of size 1000, followed by two fully‐connected layers, each containing 100 neurons and using ReLU activation functions. The Unet model is a simple 3D Unet with a three‐level encoder‐decoder structure. The encoder comprises two convolutional blocks, each consisting of two convolutional layers (kernel size = 3), followed by batch normalization and ReLU activation. The first block uses 32 filters, while the second employs 64 filters, with both blocks subsequently followed by max pooling for spatial downsampling. A bottleneck convolutional block with 128 filters further extracts high‐level features. The decoder path mirrors the encoder, using transposed convolutional upsampling layers (kernel size = 2) and skip connections to restore spatial details, with filter numbers decreasing from 128 to 64 and then 32. A final 1×1×1 convolution with a sigmoid activation generates the AC cuboid output, which is continuous between 0 and 1 and serves as a probability map.

For the model training, all LSTM models employed a mean squared error (MSE)‐based loss. Specifically, Model 0 utilized a standard MSE loss, while the loss function L of Model 1, 2, and 3 consisted of a standard MSE loss with an additional loss term to focus on the dose in the ACs.

(1) $$L=\mathrm{MSE}\left(D,\hat{D}\right)+\lambda \cdot \mathrm{MSE}\left(\mathrm{AC}⊙D,\mathrm{AC}⊙\hat{D}\right)$$

where D is the MC dose cuboid matrix, $\hat{D}$ is the predicted dose cuboid matrix, AC is the AC cuboid matrix, ⊙ represents element‐wise multiplication (Hadamard product), and λ is the weighting factor (where factors of 1, 10, and 20 were explored, with 10 found to be optimal based on validation results and used).

All Unet models used the binary cross‐entropy (BCE) loss, consistent with the AC segmentation study.^43^ The Adam optimizer was employed to minimize the corresponding loss functions of our models. The learning rate was set to $10^{-5}$ and the batch size to 4. Models were implemented based on Python 3.11.9 and Pytorch 1.13.0 with an NVIDIA RTX A6000 GPU (48 GB memory) and the training time of each model was within two days. Models with the lowest validation loss were evaluated on the test dataset.

### 2.6 Evaluation

After obtaining photon beam dose predictions from the four models, the 3D global gamma passing rates ($\gamma_{\mathrm{pr}}$ with a 2%/2 mm criteria and a threshold of 10% of the maximum dose $D_{\max}$) between the predicted dose and MC dose were evaluated per cuboid using the open‐source library PyMedphys.^44^ The Wilcoxon signed‐rank test was applied to $\gamma_{\mathrm{pr}}$ to assess statistically significant differences between the models. Additionally, for each dose cuboid, a dose mask was generated using a 15% $D_{\max}$ threshold to determine whether the dose passed through the ACs. The average $\gamma_{\mathrm{pr}}$ was then calculated for the cuboids where the dose intersected the ACs.

The Dice similarity coefficients (DSCs) between the manually contoured ACs and the Unet‐based AC auto‐segmentation on MRI were evaluated, where a threshold of 0.4, selected based on the highest validation DSC among thresholds ranging from 0.3 to 0.7 in increments of 0.1, was applied to the predicted AC to generate the binary mask. To quantify the size of ACs, we used 3D connected component analysis with 18‐connectivity—where a voxel is connected to 6 face‐sharing and 12 edge‐sharing neighbors—on both manual and Unet‐based masks to compute individual volumes, and further compared them before and after applying a 1 cc threshold, which reflects a more clinically practical override strategy.

For the single beam dose, the central axis dose profiles in beam direction (z‐axis) and the lateral dose profiles (x‐axis) in 30% and 80% of the maximum dose with corresponding relative dose differences $\varepsilon_{\mathrm{rel}}$ (%) were evaluated.

(2) $$\varepsilon_{\text{rel}}=200\frac{\left(D_{\text{pred}}-D_{\text{MC}}\right)}{\left(D_{\text{pred}}+D_{\text{MC}}\right)}\left[\%\right]$$

where $D_{\text{pred}}$ and $D_{\text{MC}}$ are the predicted and MC dose values through the central axis or lateral dose profiles.

To compare the full plan dose distributions obtained from the MC simulation and model predictions, we evaluated the 3D global gamma passing rate $\gamma_{\mathrm{pr}}$ (2%/2 mm, $D>10\%D_{\max}$), along with the dose volume histogram (DVH) and DVH indices for typical structures, including PTV (D_2%_, D_95%_), bladder (D_2%_, V_60Gy_ and V_65Gy_) and rectum (D_2%_, V_50Gy_, V_60Gy_, V_65Gy_).

The model prediction runtime includes the cuboid extraction time and the model inference time (LSTM and Unet). All computation measurements were carried out on a workstation equipped with an Intel(R) Xeon(R) Gold 6354 3.00 GHz CPU and an NVIDIA RTX A6000 GPU.

## 3 RESULTS

### 3.1 Beam dose prediction results of Models 0, 1, 2, and 3

**FIGURE 4:**
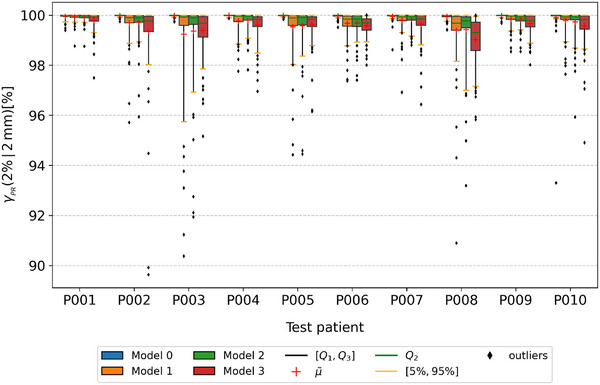
Patient specific boxplots for $\gamma_{\mathrm{pr}}$ of predicted dose cuboids from Models 0, 1, 2, and 3 in the test dataset. The red cross denotes the mean and the green line denotes the median value. The boxes denote the range between 25th and 75th percentiles. The orange whiskers denote the 5th and 95th percentiles, while the black points denote outliers outside of that range.

To evaluate the overall performance of the four models, the patient specific boxplots of $\gamma_{\mathrm{pr}}$ of predicted doses in the test dataset are shown in Figure 4. Although the MRI input‐based models (Models 1, 2, 3) have slightly worse performances than the CT input‐based model (Model 0), Models 1, 2, 3 can still achieve $\gamma_{\mathrm{pr}}$ 95th percentiles above 95%. Model 2 (using a separately trained Unet to predict AC) performed comparably to Model 1 (using ground truth AC). Table 1 presents the mean and worst $\gamma_{\mathrm{pr}}$ values of predicted doses from the four models, along with the mean DSC metrics of predicted ACs from the Unet of models 2 and 3 in the test dataset. The mean $\gamma_{\mathrm{pr}}$ of all models in the test dataset was higher than 99.5% and the mean DSC of the AC segmentation above 0.6. Table 2 presents the mean, standard deviation (SD), and range of AC volumes for both manual contours and Unet predictions in the test dataset. On average, each test patient had 125 manually contoured ACs, which was reduced to 5 after applying the 1 cc volume threshold. The mean DSC after filtering was 0.65. In addition, $\gamma_{\mathrm{pr}}$ comparisons using the Wilcoxon signed‐rank test (p<0.01) revealed statistically significant differences between Model 0 (CT to dose) and Model 1 (MR with manual AC to dose), Model 1 and Model 2 (MR with Unet‐predicted AC to dose, trained separately), as well as Model 2 and Model 3 (MR with Unet‐predicted AC to dose, trained jointly).

**TABLE 1:** Comparison of $\gamma_{\text{pr}}$ and DSC values across four models in the test dataset.

	$\gamma_{\text{pr}}$ (total)	$\gamma_{\text{pr}}$ (dose through AC)	DSC
Model	mean±SD (min)	mean±SD (min)	mean±SD
Model 0	99.96±0.22 (93.30)	99.94±0.25 (93.30)	\
Model 1	99.69±0.77 (90.37)	99.68±0.81 (94.42)	\
Model 2	99.68±0.87 (91.93)	99.66±0.91 (94.45)	0.62±0.20
Model 3	99.53±0.78 (89.63)	99.39±1.10 (89.63)	0.60±0.21

Abbreviations: AC, air cavities; DSC, dice similarity coefficient; SD, standard deviation.

**TABLE 2:** The mean, SD, and range of the volume of ACs in the test dataset.

Dataset	Filtering	Mean±SD (${\mathrm{cm}}^{3}$)	Range (${\mathrm{cm}}^{3}$)
Manual AC	None	0.25±1.37	0.01–21.42
Manual AC	≥1 ${\mathrm{cm}}^{3}$	5.47±4.89	1.00–21.42
Unet AC	None	0.64±2.71	0.01–44.65
Unet AC	≥1 ${\mathrm{cm}}^{3}$	6.69±6.96	1.00–44.65

Abbreviations: AC, air cavities; SD, standard deviation.

**FIGURE 5:**
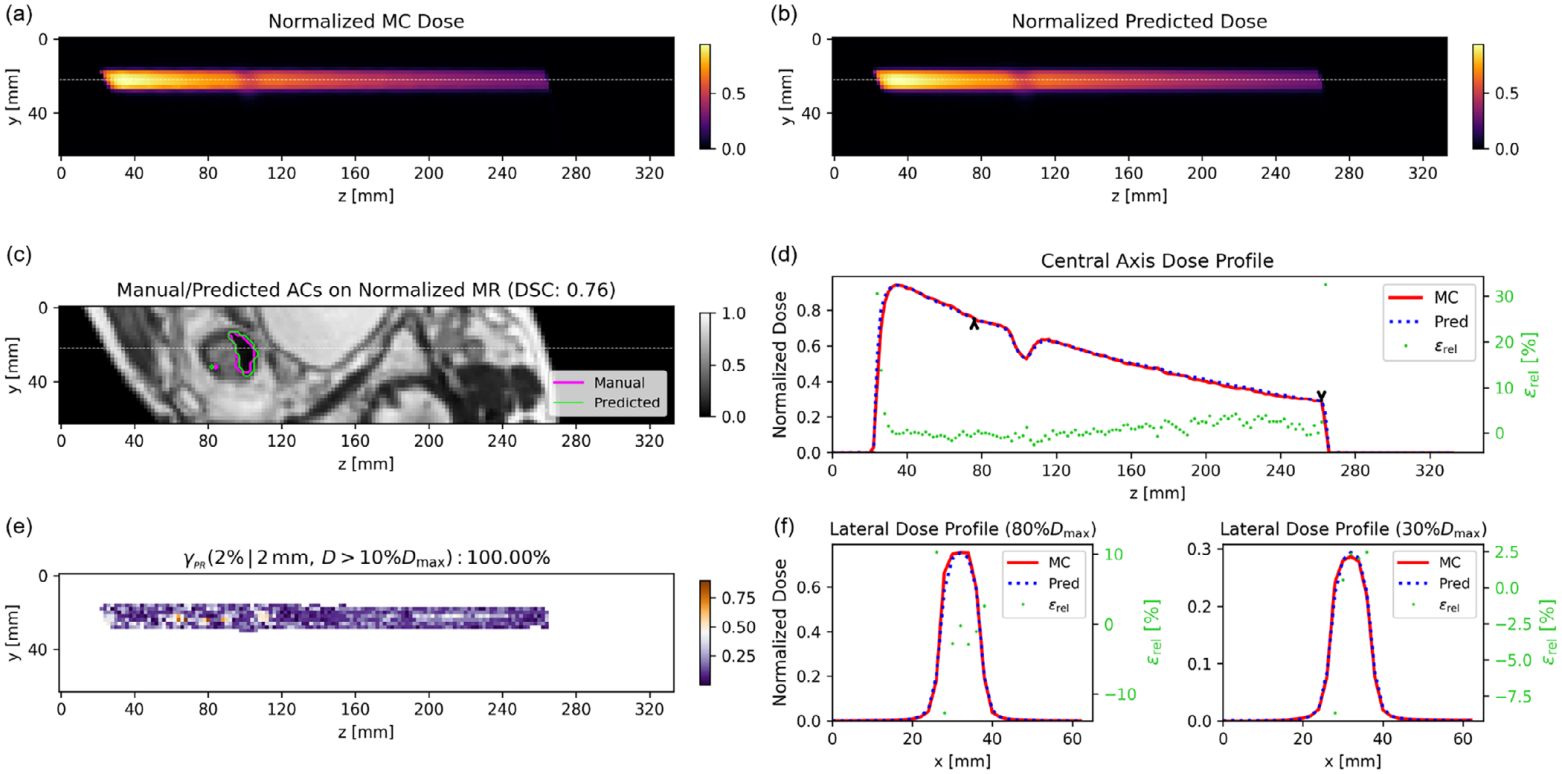
A nominal case predicted by Model 2 (dose through AC, from P001 in Figure 4): *yz* slices of normalized (a) MC and (b) predicted dose, (c) manual and predicted AC on MRI, (d) the central axis depth dose profiles of normalized MC and predicted dose, (e) Γ (2%/2 mm) map of central dose plane, and (f) the lateral dose profiles of normalized MC and predicted dose with the relative dose differences. The central depth dose profile is through the gray dashed line and the positions of the two lateral dose profiles are indicated by two black arrows in the central axis dose profile.

**FIGURE 6:**
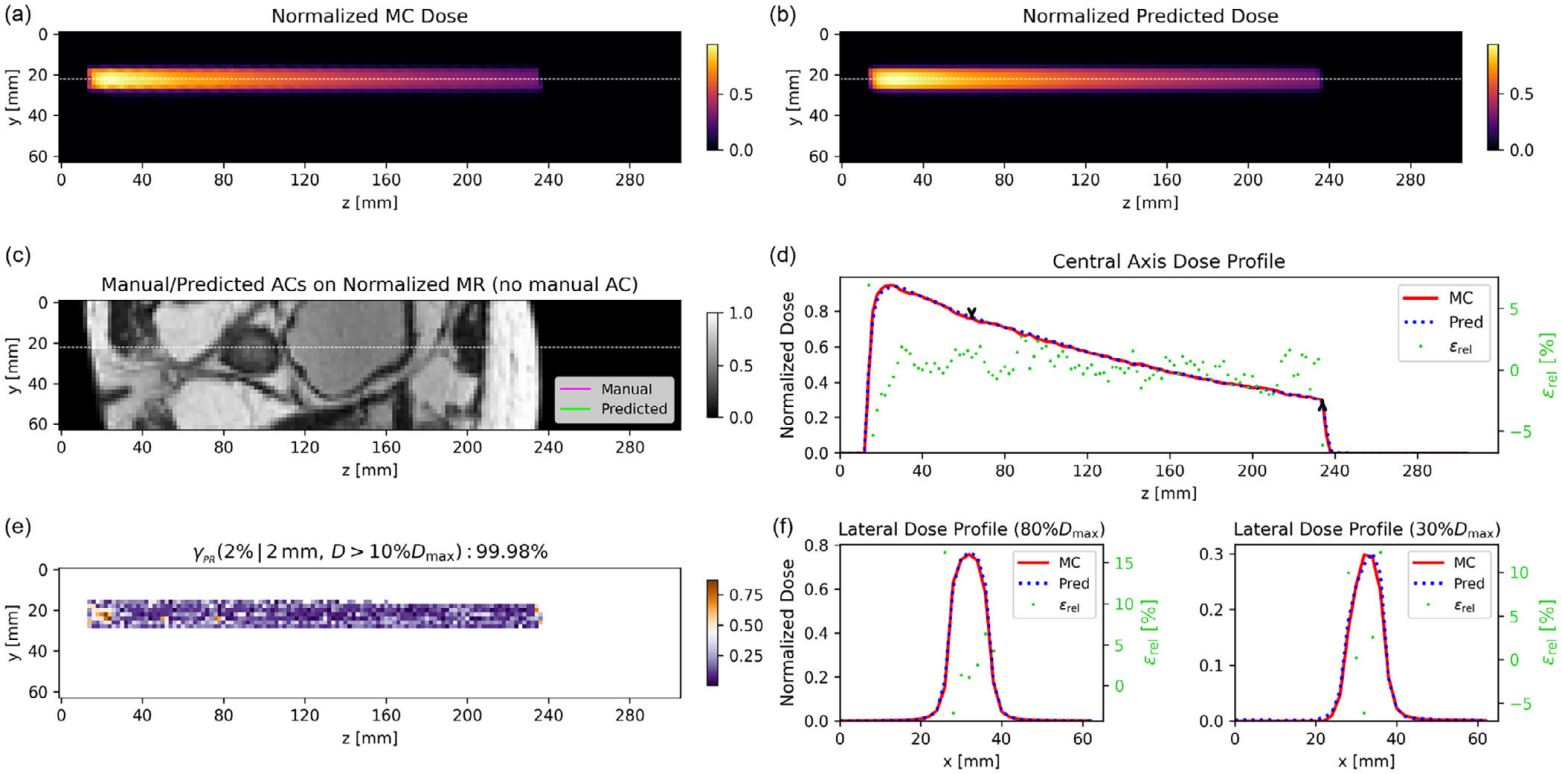
A nominal case predicted by Model 2 (dose not through AC, from P004 in Figure 4): *yz* slices of normalized (a) MC and (b) predicted dose, (c) manual and predicted AC on MRI, (d) the central axis depth dose profiles of normalized MC and predicted dose, (e) Γ (2%/2 mm) map of central dose plane, and (f) the lateral dose profiles of normalized MC and predicted dose with the relative dose differences. The central depth dose profile is through the gray dashed line and the positions of the two lateral dose profiles are indicated by two black arrows in the central axis dose profile.

**FIGURE 7:**
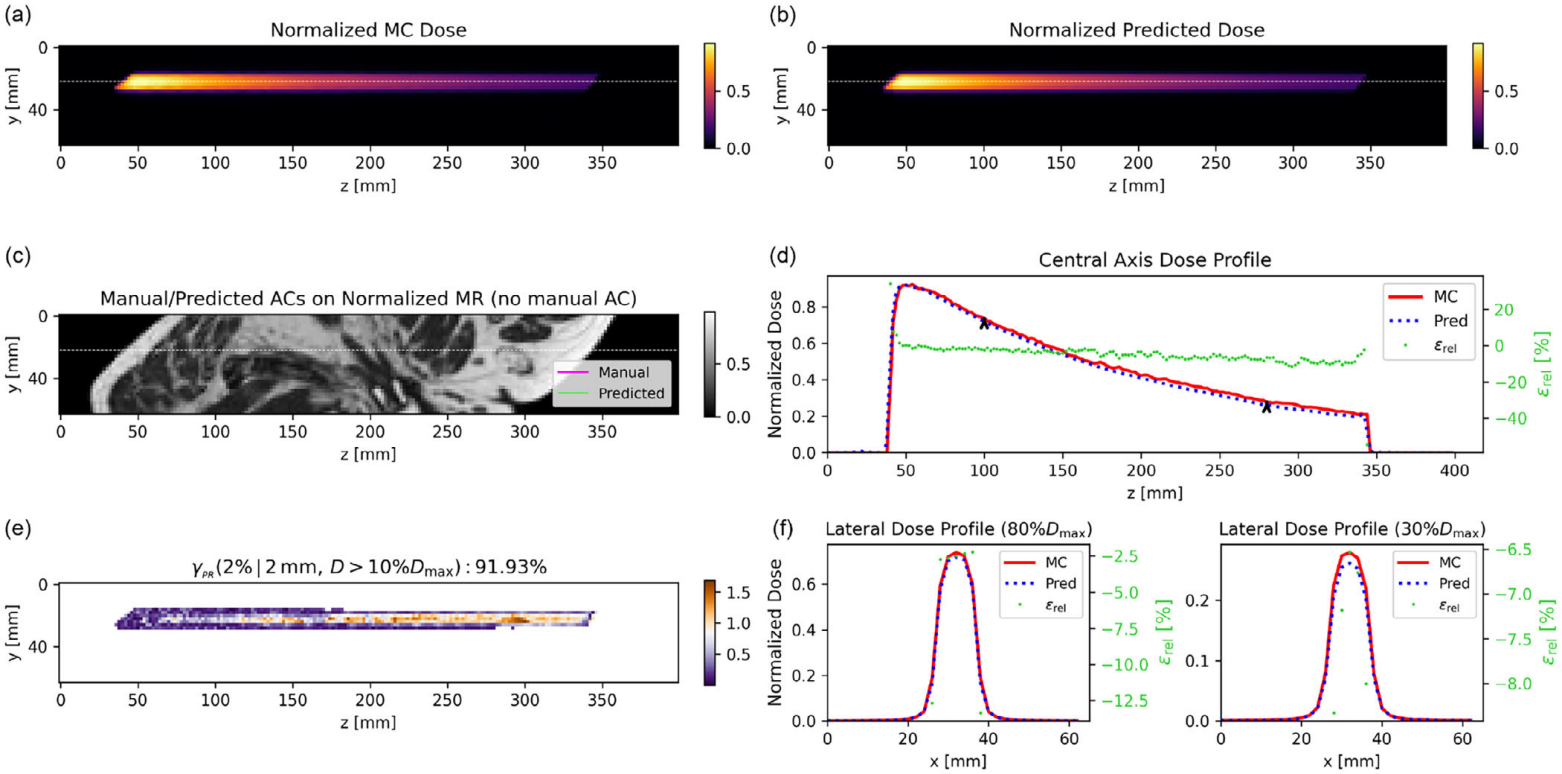
The worst case predicted by Model 2 (from P003 in Figure 4): *yz* slices of normalized (a) MC and (b) predicted dose, (c) manual and predicted AC on MRI, (d) the central axis depth dose profiles of normalized MC and predicted dose, (e) Γ (2%/2 mm) map of central dose plane, and (f) the lateral dose profiles of normalized MC and predicted dose with the relative dose differences. The central depth dose profile is through the gray dashed line and the positions of the two lateral dose profiles are indicated by two black arrows in the central axis dose profile.

Since Model 2 had $\gamma_{\mathrm{pr}}$ which were similar or better than Model 3, we selected Model 2 for the subsequent analysis. Model 1 was not considered due to the impossibility of manually delineating AC in real‐time. For better visualization, beam dose prediction example cases were cropped to focus on the main dose region. Figure 5 presents a nominal case of dose propagation through AC, as predicted by Model 2 on the test dataset. The AC is automatically contoured from the MRI cuboid, achieving a DSC of 0.76. Subsequently, the LSTM model predicts the dose distribution with a $\gamma_{\mathrm{pr}}$ passing rate of 100%. The central axis depth dose profile and two lateral dose profiles (at 30% and 80% of the maximum dose) for the predicted and MC dose matched well. Additionally, Model 2 accurately captured the dose fall‐off within the AC. Figure 6 presents a nominal case where the dose does not pass through an AC, as predicted by Model 2 on the test dataset. The central axis depth dose profiles of predicted and MC dose exhibited strong agreement, with relative dose differences $\varepsilon_{\mathrm{rel}}$ smaller than 5% and a $\gamma_{\mathrm{pr}}$ of 99.98%. The two lateral dose profiles also demonstrated good consistency. Figure 7 shows the worst predicted case among the 10 test patients, which corresponds to a case where the dose does not pass through an AC. The central axis depth and two lateral dose profiles indicate that the predicted dose is lower than the MC dose, with a corresponding $\gamma_{\mathrm{pr}}$ of 91.93%.

**FIGURE 8:**
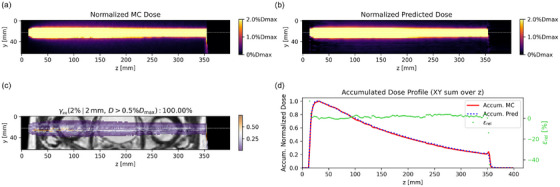
A nominal case predicted by Model 2 highlighting the low‐dose region: *yz* slices of normalized (a) MC and (b) predicted dose, (c) Γ (2%/2 mm) map of central dose plane on MRI, (d) the normalized laterally accumulated dose profiles of MC dose and predicted dose with the relative differences.

Due to the ERE, we also presented a single‐beam dose prediction example, highlighting the model's performance in the low‐dose region, as shown in Figure 8. The colorbars for the MC and predicted dose were normalized to $2\%D_{\max}$ of the MC dose as the upper limit and the prediction accuracy was assessed using the gamma passing rate $\gamma_{\mathrm{pr}}$ (2%/2 mm, $D>0.5\%D_{\max}$). From the dose distribution and the laterally accumulated dose profile comparison at the surface area, Model 2 exhibited limitations in accurately predicting the ERE near the patient surface.

### 3.2 Dose prediction results of five plans using Model 2

**FIGURE 9:**
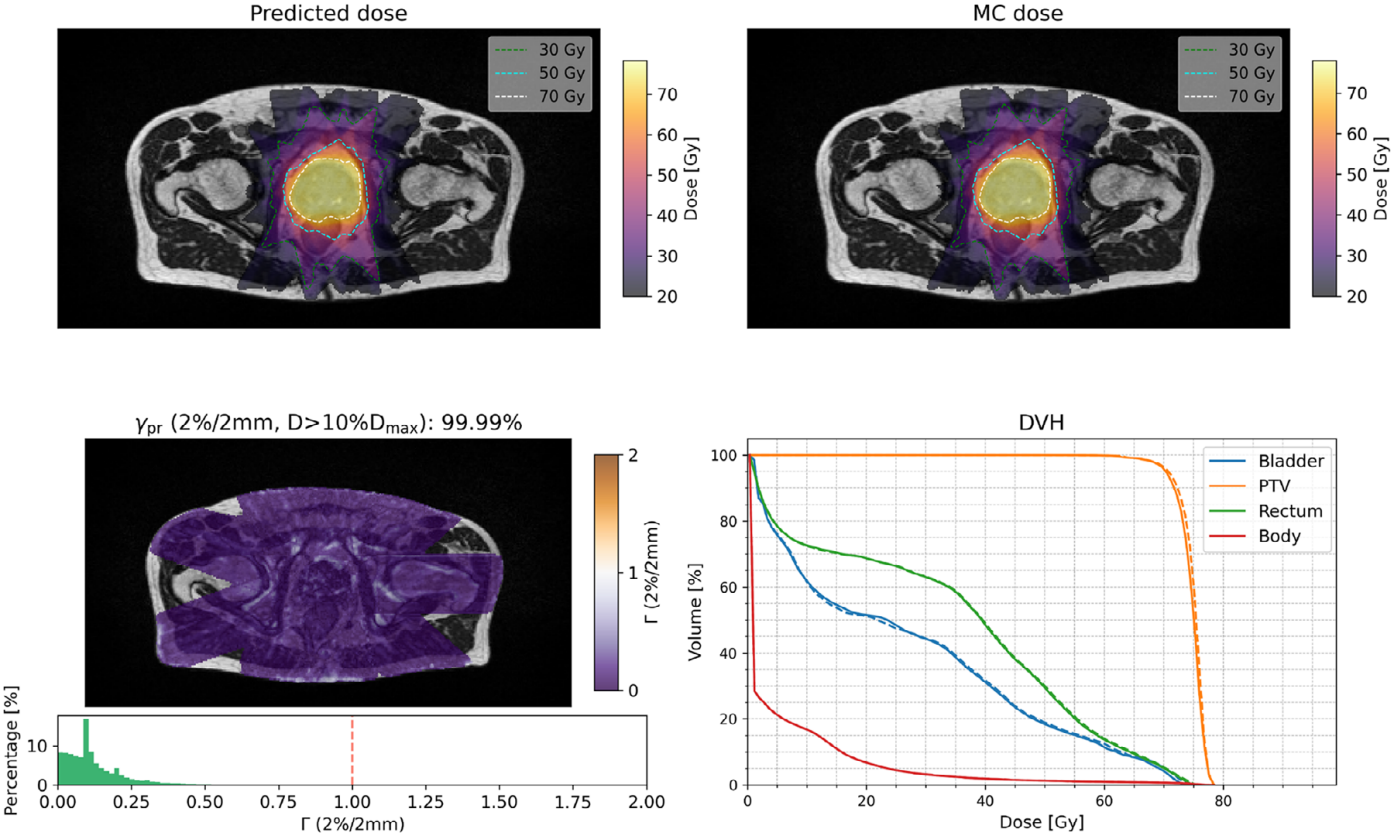
Transverse slice of the (a) predicted dose, (b) MC dose, both with the 30, 50, and 70 Gy isodose lines, (c) Γ (2%/2 mm) map with the corresponding Γ index histogram, and (d) DVH (predicted dose solid line and MC dose as dashed line) for Plan 1 (from P002). MC, Monte Carlo; DVH, dose volume histogram.

**FIGURE 10:**
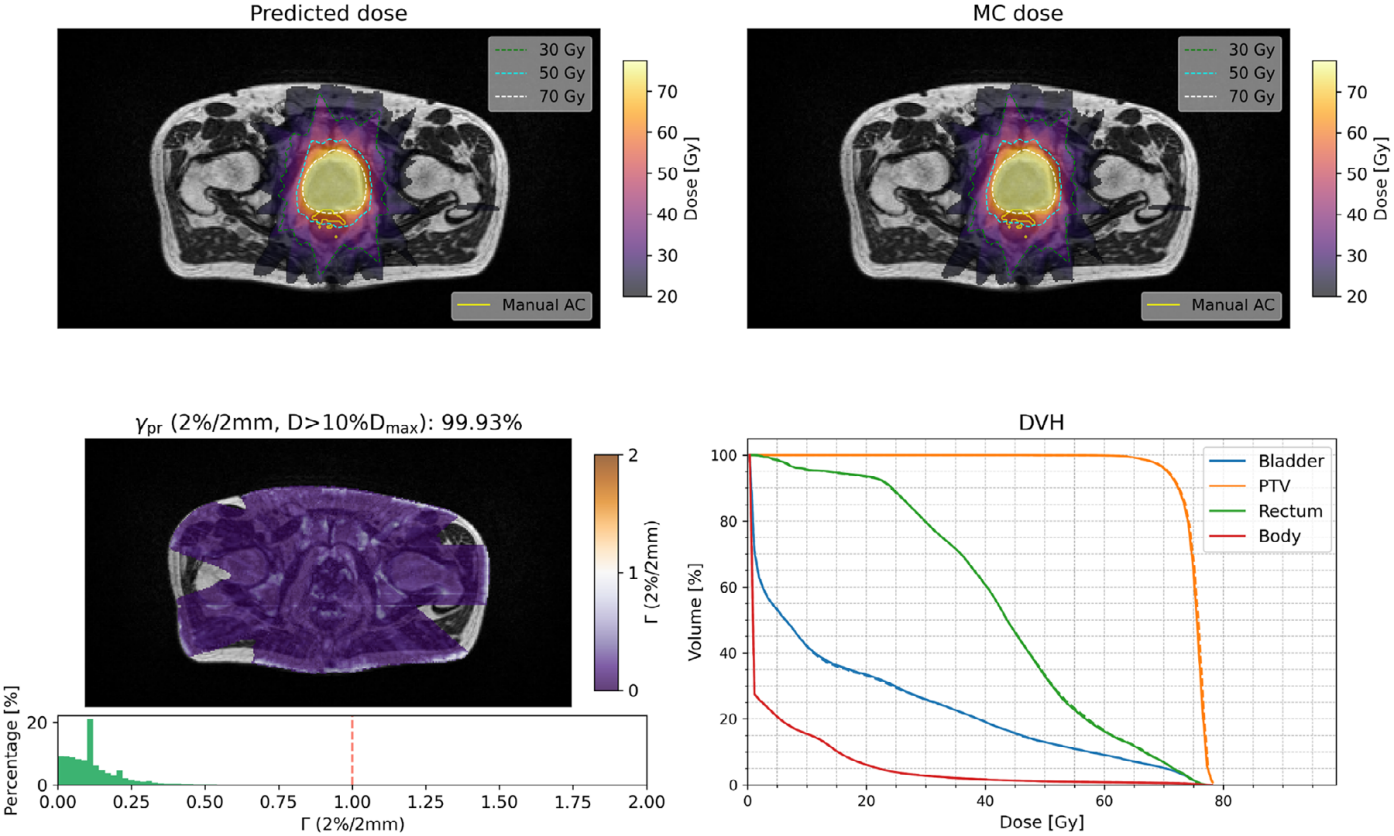
Transverse slice of the (a) predicted dose, (b) MC dose, both with the 30, 50, and 70 Gy isodose lines, (c) Γ (2%/2 mm) map with the corresponding Γ index histogram, and (d) DVH (predicted dose solid line and MC dose as dashed line) for Plan 2 (from P006). An AC is visible in the rectal area under the PTV on the MRI. MC, Monte Carlo; DVH, dose volume histogram; PTV, planning training volume.

Figures 9 and 10 show the comparison of the plan dose distributions predicted from Model 2 and MC simulations for plans 1 (from P002) and 2 (from P006, with an AC near the PTV on MRI), respectively, with the comparisons for plans 3–5 provided in Figures S1– S3. The $\gamma_{\mathrm{pr}}$ values of the two plans were 99.99% and 99.93% respectively. The DVH curves for the predicted and MC doses from two cases are close to each other in the low and high dose levels for the evaluated PTV and organ‐at‐risk structures. Table 3 presents the corresponding DVH indices for the five test plans, with the largest observed differences being less than 1.0 Gy and 1.2%. Table 4 shows the $\gamma_{\mathrm{pr}}$ comparision of five full plans with 2%/2 mm, 1%/2 mm, 1%/ 1mm criteria with the threshold of $D>10\%D_{\max}$.

**TABLE 3:** DVH indices and differences (prediction‐MC) of five plan dose distributions from Model 2 and MC simulation. Prescription dose 74 Gy.

		PTV		Bladder			Rectum			
		D_2%_ (Gy)	D_95%_ (Gy)	D_2%_ (Gy)	V_60Gy_ (%)	V_65Gy_ (%)	D_2%_ (Gy)	V_50Gy_ (%)	V_60Gy_ (%)	V_65Gy_ (%)
Plan 1 (P002)	Model 2	77.3	70.0	71.2	11.0	8.2	72.4	28.7	13.3	9.1
	MC	77.3	70.4	72.1	12.1	8.5	72.8	28.7	13.6	9.4
	Difference	0.0	−0.4	−0.9	−1.1	−0.3	−0.4	0.0	−0.3	−0.3
Plan 2 (P006)	Model 2	77.4	70.2	74.1	9.0	6.9	74.1	32.0	15.8	11.4
	MC	77.4	70.4	74.0	8.9	6.9	73.9	32.0	16.0	11.6
	Difference	0.0	−0.2	0.1	0.1	0.0	0.2	0.0	−0.2	−0.2
Plan 3 (P005)	Model 2	77.0	69.9	71.9	10.0	7.5	70.7	12.9	8.4	5.6
	MC	77.5	70.4	72.1	10.1	7.9	71.2	12.9	8.8	6.0
	Difference	−0.5	−0.5	−0.2	−0.1	−0.4	−0.5	0.0	−0.4	−0.4
Plan 4 (P008)	Model 2	79.0	70.2	75.8	13.6	10.9	70.2	13.8	8.1	5.2
	MC	78.9	70.4	76.0	13.9	11.4	70.0	13.8	8.1	5.0
	Difference	−0.1	−0.2	−0.2	−0.3	−0.5	0.2	0.0	0.0	0.2
Plan 5 (P009)	Model 2	78.3	70.3	76.1	25.4	20.6	71.7	17.0	11.2	7.8
	MC	78.6	70.4	75.9	25.4	20.7	71.8	17.0	11.3	8.0
	Difference	−0.3	−0.1	0.2	0.0	−0.1	−0.1	0.0	−0.1	−0.2

Abbreviations: DVH, dose volume histogram; MC, Monte Carlo; PTV, planning training volume.

**TABLE 4:** $\gamma_{\mathrm{pr}}$ comparison of five full plans with 2%/2mm, 1%/2mm, and 1%/1mm criteria in the $D>10\%D_{\max}$ region.

	$\gamma_{\mathrm{pr}}$ (2%/2mm)	$\gamma_{\mathrm{pr}}$ (1%/2mm)	$\gamma_{\mathrm{pr}}$ (1%/1mm)
Plan	value (%)	value (%)	value (%)
Plan 1 (P002)	99.99	99.67	98.81
Plan 2 (P006)	99.93	99.49	97.97
Plan 3 (P005)	99.89	99.17	97.38
Plan 4 (P008)	99.80	99.36	97.91
Plan 5 (P009)	99.96	99.71	98.56

### 3.3 Model prediction runtimes

The average extraction time for an MRI cuboid was about 43 ms, and the averaged inference time of the LSTM and Unet model for a single photon dose cuboid was around 7 and 5 ms, respectively. In total, the prediction time of Model 2 was around 60 ms, with a GPU memory requirement of less than 2 GB for inference.

## 4 DISCUSSION

In this study, we introduced a DL‐based synthetic‐CT‐free photon dose calculation method, integrating LSTM and Unet networks. In these initial results, focusing on prostate radiotherapy under a 0.35 T magnetic field, we demonstrated that our approach effectively models the mapping between MRI and photon beam dose distributions simulated on the corrected deformed CT. Although using MRI as model input results in a slight reduction in accuracy compared to the model that directly uses CT (as shown in Figure 4), our MRI‐based model (Model 2) can still achieve a mean gamma passing rate $\gamma_{\mathrm{pr}}$ (2%/2 mm, $D>10\%D_{\max}$) higher than 99.5% in the single‐beam test. Besides, both the DVH analysis and gamma passing rate $\gamma_{\mathrm{pr}}$ (2%/2 mm, $D>10\%D_{\max}$) demonstrated high agreement in the full‐plan evaluation, suggesting the feasibility of performing DL dose calculations directly on MRI. In contrast to previously published DL‐based dose calculation methods that rely on CT‐based input, end‐to‐end dose calculation on MRI can bypass the time‐consuming process of generating 3D synthetic CT. Additionally, the proposed framework can consider AC variations near the prostate region in real‐time.

Model 3, trained with a joint multi‐task loss, performed slightly worse than the two single‐task sub‐models (Model 2). The heterogeneous objectives, using BCE for voxel‐wise binary classification and MSE for regression, might have caused loss‐scale imbalance that impeded joint optimization. Given that the separately trained sub‐models of Model 2 already demonstrated satisfactory performance, we did not further pursue more complex multi‐task strategies in this study. For the worst predicted beam dose case from Model 2, we have two potential hypotheses for the reduced prediction accuracy: First, the subtle spatial misalignment between the input MR and the ground‐truth corrected CT at the beam entrance region may have contributed to the error, as shown in the Figure S4. Due to the dose values outside the body mask derived from the MRI being set to zero, such misalignment in high‐dose‐gradient regions may lead to dose over‐ or underestimation and affect subsequent dose propagation. Second, we cannot exclude the possibility that residual errors from the deformable registration process may introduce spatial mismatches that degraded prediction accuracy. Using a fused CT/MR body contour or a water equivalent correction for the corrected CT may be a more robust approach for such cases. In addition, although our LSTM model exhibited limitations in accurately predicting the ERE near the patient surface, for multi‐field plans (Figures 9 and 10) on low‐field (0.35 T) MR‐Linacs, the cumulative dose contributions from multiple beam angles inherently attenuate localized dose enhancements caused by ERE. An additional loss function targeting surface dose regions and more advanced neural network architectures will be explored to address this issue in future studies.

Clinically, MRI is affected by low‐frequency intensity inhomogeneity changes, commonly known as bias field artifacts.^45^ These artifacts result from various factors related to image acquisition, including coil positioning, coil sensitivities, magnetic field inhomogeneities, and patient‐related factors. Correcting these low‐frequency artifacts may help improve our DL model's performance. To assess the impact of bias field artifacts, we applied the N4 bias field correction algorithm using the same parameters as those used in another DL segmentation study^46^ on our 0.35 T MRI and compared the results with the uncorrected MRI using Model 2. Consistent with the findings in that study,^46^ we observed that bias field correction did not enhance the DL dose prediction outputs. Our DL model is robust to the variations caused by bias field artifacts, suggesting that DL dose calculation on MRI can be performed accurately without the need for bias field corrections.

The MR scanner used in this study has a magnetic field strength of 0.35 T and employs the bSSFP sequence, with MC dose simulation also conducted under an idealized 0.35 T magnetic field and using a generic 6 MV photon source. It may be worthwhile to explore DL dose calculation using 1.5 T MRI and under the 1.5 T magnetic field from an Elekta Unity MR‐Linac.^2^ While the improved MRI image resolution and signal‐to‐noise ratio from a stronger magnetic field might enhance DL dose calculation accuracy, the associated stronger magnetic dose effects might also complicate DL dose modeling. In addition, specialized MR sequences, such as Cartesian 3D FLASH or 3D tFE,^43^ could be utilized to further enhance the accuracy of DL‐based AC segmentation in the abdominopelvic region. Our models achieved an average DSC of approximately 0.6, which improved to around 0.65 after filtering out small AC contour region with the volume less than 1 cc, suggesting potential for further improvement with a more suitable MRI sequence as demonstrated in another study.^43^ Besides, instead of performing AC segmentation across the entire patient anatomy, we opted to apply AC segmentation within a cuboid region that specifically targets areas relevant to dose calculation, thereby improving computational efficiency.

Although the dosimetric impact of ACs is often considered negligible in multi‐beam or arc therapy due to dose averaging and scattering of large field beams for prostate patients, a rectum IMRT study has shown that air pockets near or within the target volume can introduce non‐negligible local dose perturbations under the magnetic field.^47^ Moreover, air pockets may vary in shape and position during and between fractions, introducing residual uncertainties.^48^ For the dose effect of the ACs, small fields present a clearer observation of the dose reduction in the air cavity, where the phenomenon can be masked by the re‐establishment of charged particle equilibrium from larger fields. For these initial proof‐of‐concept results, we simulated all the photon beams with a uniform $1\times 1\;{\mathrm{cm}}^{2}$ field size and optimized five nine‐field plans for testing. This configuration can not only generate arbitrary fluence maps using optimized weights, but also enables a clearer evaluation of local dose deviations and supports the development of segmentation‐based correction strategies. The network was only specifically trained for prostate treatment sites. The ultimate goal of DL photon dose calculation on MRI is to accurately compute dose distributions across multiple treatment sites with varying multileaf collimator (MLC) shapes. As the current framework lacks dedicated modeling for MLC MC simulation, incorporating MLC information into the DL model will be part of the next stage. MLC segment BEV projection methods, similar to those used in CT‐based DL dose calculation,^22^, ^23^ could be applied in our future studies.

This study focused and is therefore limited to prostate cases. For more complex anatomical structures outside the prostate region, such as the abdomen and lungs, further exploration remains both essential and challenging. In the abdominal region, the motion of organs such as the intestines, stomach, liver, and kidneys due to respiration and peristalsis poses greater challenges for deformable CT‐MRI registration. Furthermore, the presence of larger and more dynamic ACs within multiple gas‐containing organs, including the intestines, stomach, duodenum, and rectum, further increases the complexity of AC corrections compared to prostate. In the lung region, CT‐MRI pair data exhibit significant discrepancies due to the inherently low MR signal in lung tissue (resulting from low proton density), the presence of small structures such as the bronchi, and MRI distortions, including susceptibility and motion artifacts. Additionally, manual AC corrections in the lung are often impractical, particularly in the bronchial areas. Synthetic CT generation from MRI also encounters similar challenges in lung cases, as indicated by the mean $\gamma_{\mathrm{pr}}$ (2%/2 mm, $D>10\%D_{\max}$) values, which ranged approximately 95% –96% for lung plan cases, compared to over 98% for abdominal and pelvic plan cases.^49^ Accurately obtaining paired CT‐MRI data labels is crucial, and the development of integrated CT‐MRI scanners^50^ may offer a promising solution to these challenges.

Inspired by the SynthRAD2023 Grand Challenge,^14^, ^15^ organizing a synthetic‐CT‐free dose calculation challenge, incorporating paired CT‐MRI data, plan information and corresponding time‐consuming MC dose labels from multiple sites, presents a compelling direction for future research. Instead of calculating dose on DL‐based synthetic CTs, a more direct comparison between dose predictions from MRI and MC dose from labeled CT could be achieved. End‐to‐end MRI‐based dose calculation would also enable intra‐fractional plan adaptation based on 3D time‐resolved MRI in the context of real‐time motion management.^8^ Finally, although our current DL models, based on simple LSTM and Unet architectures, have demonstrated promising performance in prostate cases, organizing such challenges to compare the performance of different DL models and training strategies, such as Unet‐based methods^22^, ^24^, ^25^, ^26^, ^27^ and LSTM/transformer‐based methods under BEV,^23^, ^29^, ^30^ would be valuable. Such a competition could provide valuable insights into the clinical applications of DL‐based dose calculation.

## 5 CONCLUSION

We proposed a DL‐based dose calculation framework that acts directly on MRI and demonstrated its feasibility in this proof‐of‐concept study for prostate cases. This approach has the potential to simplify procedures for MRI‐only workflows and could be beneficial for real‐time plan adaptation by omitting synthetic CT generation.

## CONFLICT OF INTEREST STATEMENT

The Department of Radiation Oncology of the LMU University Hospital Munich has research agreements with Brainlab, Elekta and ViewRay.

## Supporting information

Supporting Information

## Data Availability

The data cannot be made publicly available upon publication because they contain sensitive personal information. The data that support the findings of this study are available upon reasonable request from the authors.
