# Supplementary material for: Deep learning‐based synthetic‐CT‐free photon dose calculation in MR‐guided radiotherapy: A proof‐of‐concept study
Source: Med Phys. 2025 Nov 4;52(11):e70106. doi: 10.1002/mp.70106 (PMC12584934; doi:10.1002/mp.70106)
Supplement: Supplementary file 1 — Supporting Information [file MP-52-0-s001.doc]

**Supporting Material**


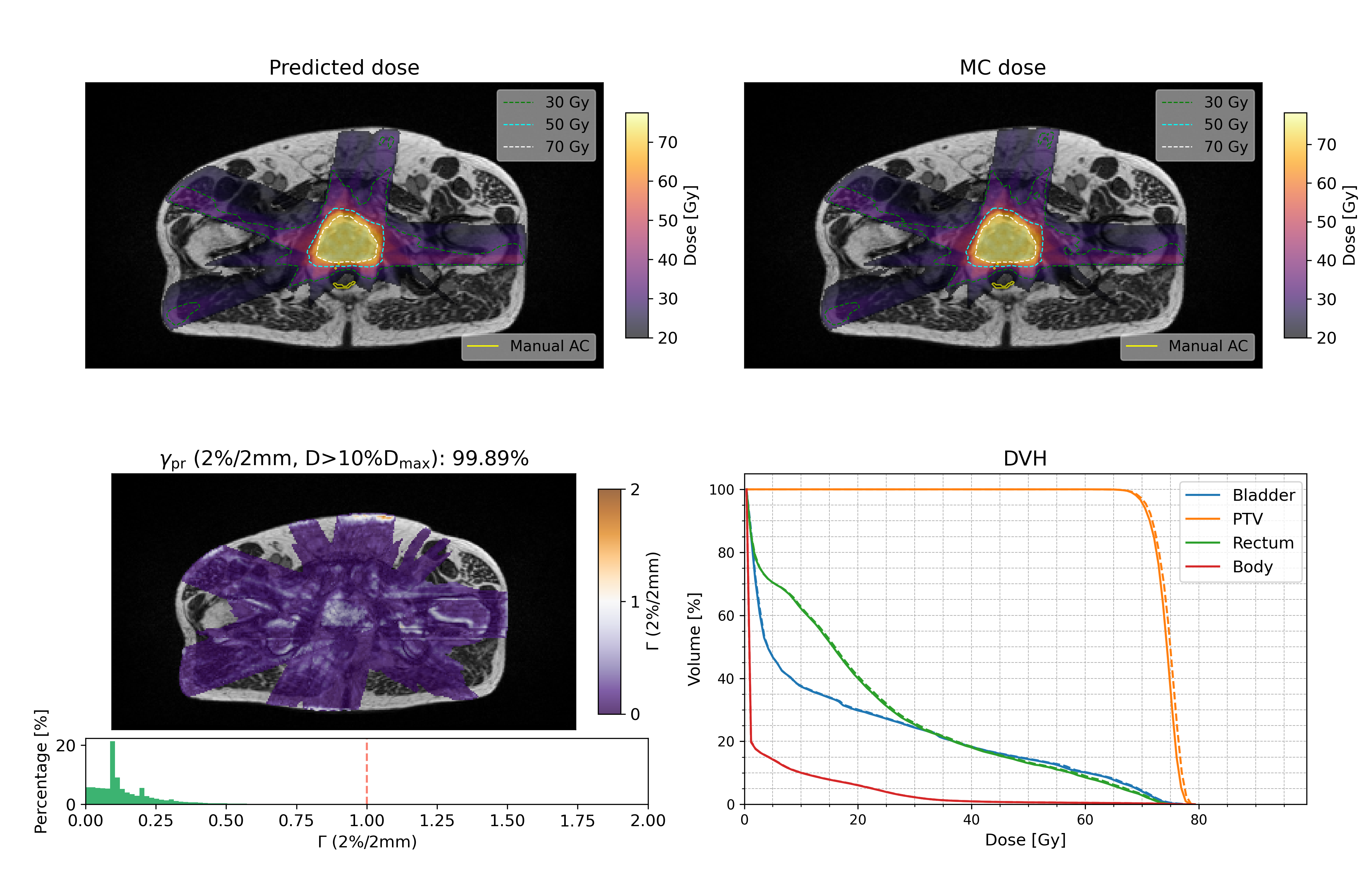


Figure S1. Transverse slice of the (a) predicted dose, (b) MC dose, both with the 30, 50, and 70 Gy isodose lines, (c) Γ (2%/2 mm) index map with the corresponding Γ index histogram, and (d) DVH (predicted dose solid line and MC dose as dashed line) for Plan 3 (from P005).


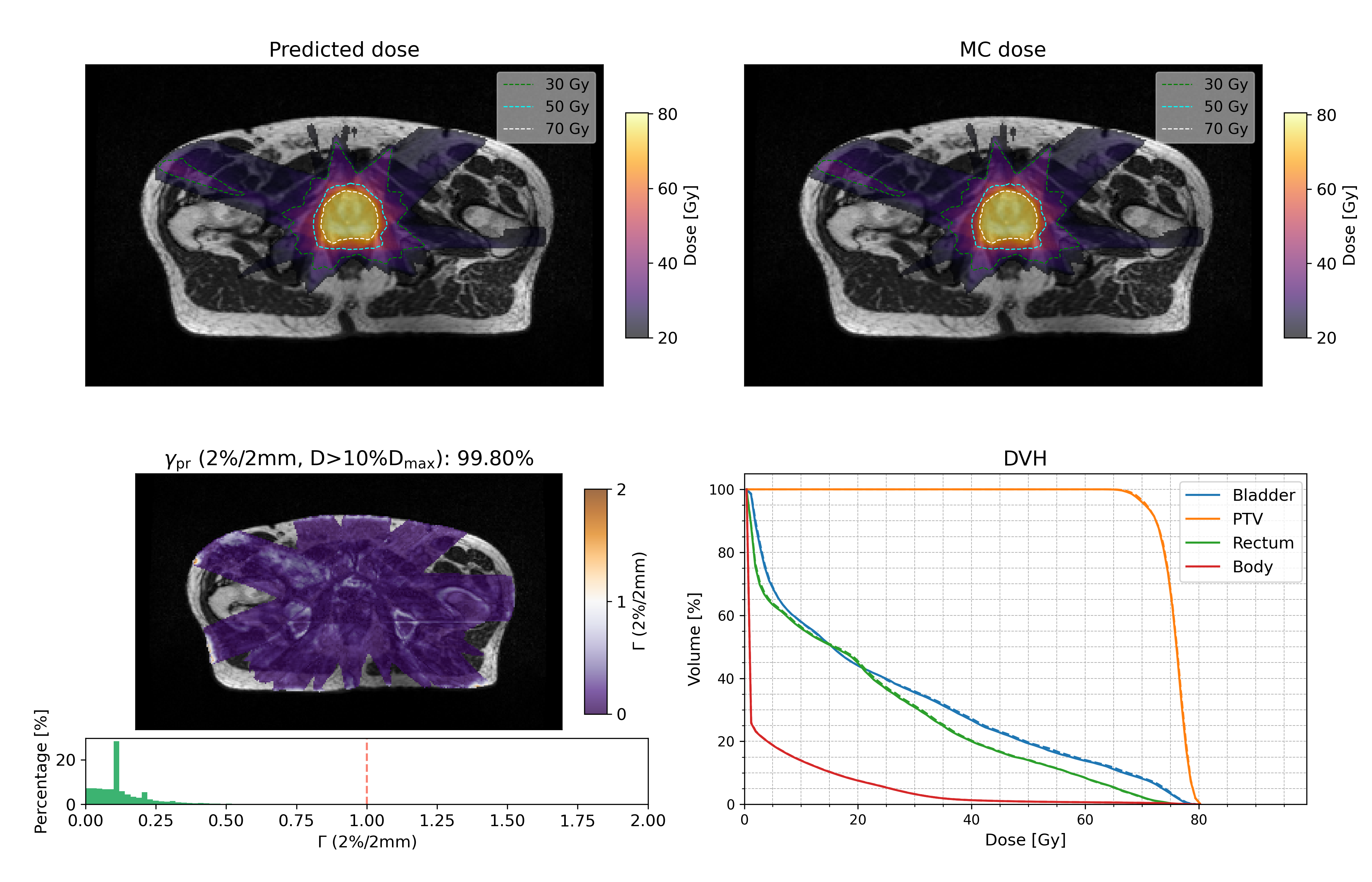


Figure S2. Transverse slice of the (a) predicted dose, (b) MC dose, both with the 30, 50, and 70 Gy isodose lines, (c) Γ (2%/2 mm) index map with the corresponding Γ index histogram, and (d) DVH (predicted dose solid line and MC dose as dashed line) for Plan 4 (from P008).


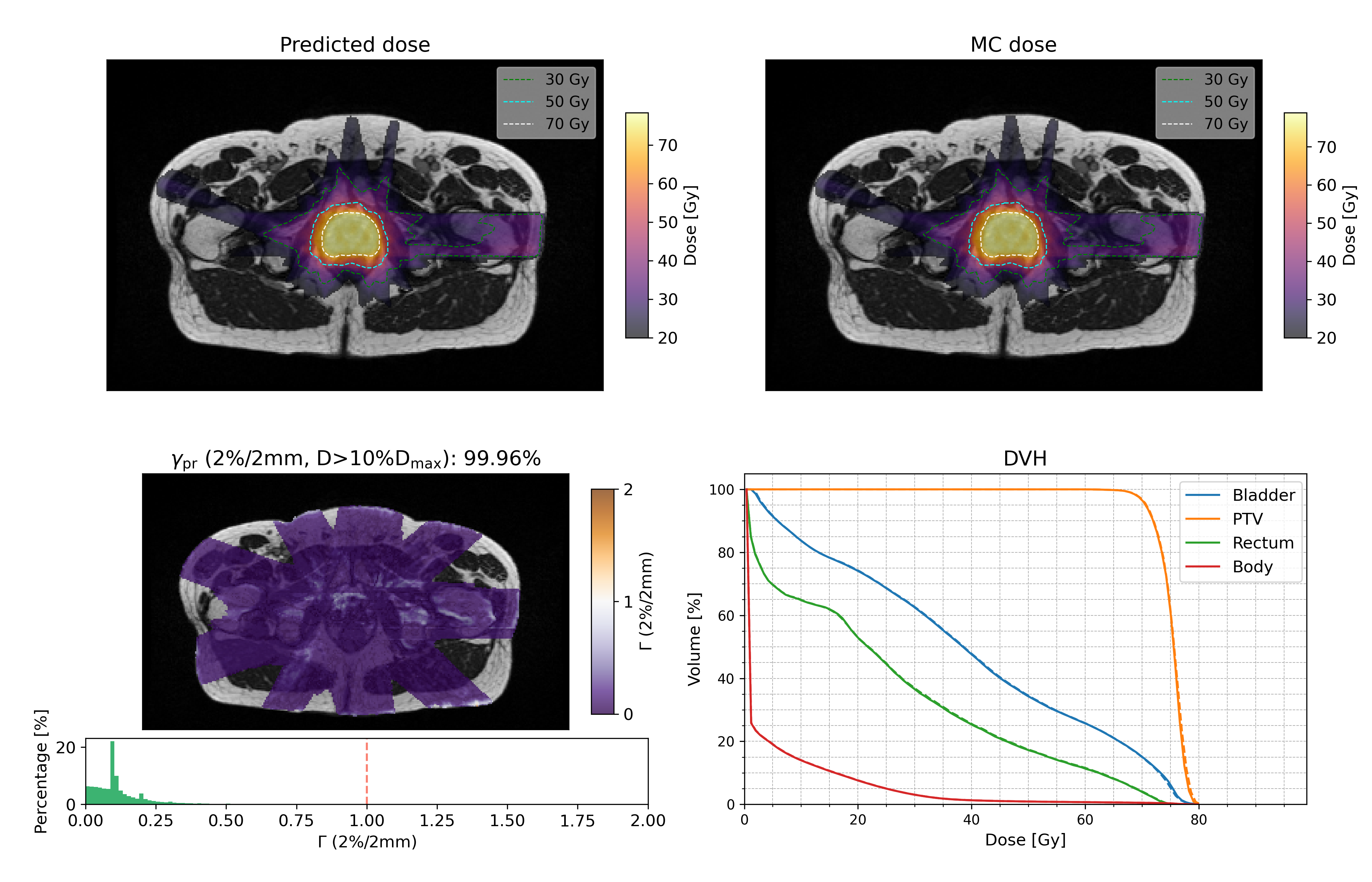


Figure S3. Transverse slice of the (a) predicted dose, (b) MC dose, both with the 30, 50, and 70 Gy isodose lines, (c) Γ (2%/2 mm) index map with the corresponding Γ index histogram, and (d) DVH (predicted dose solid line and MC dose as dashed line) for Plan 5 (from P009).


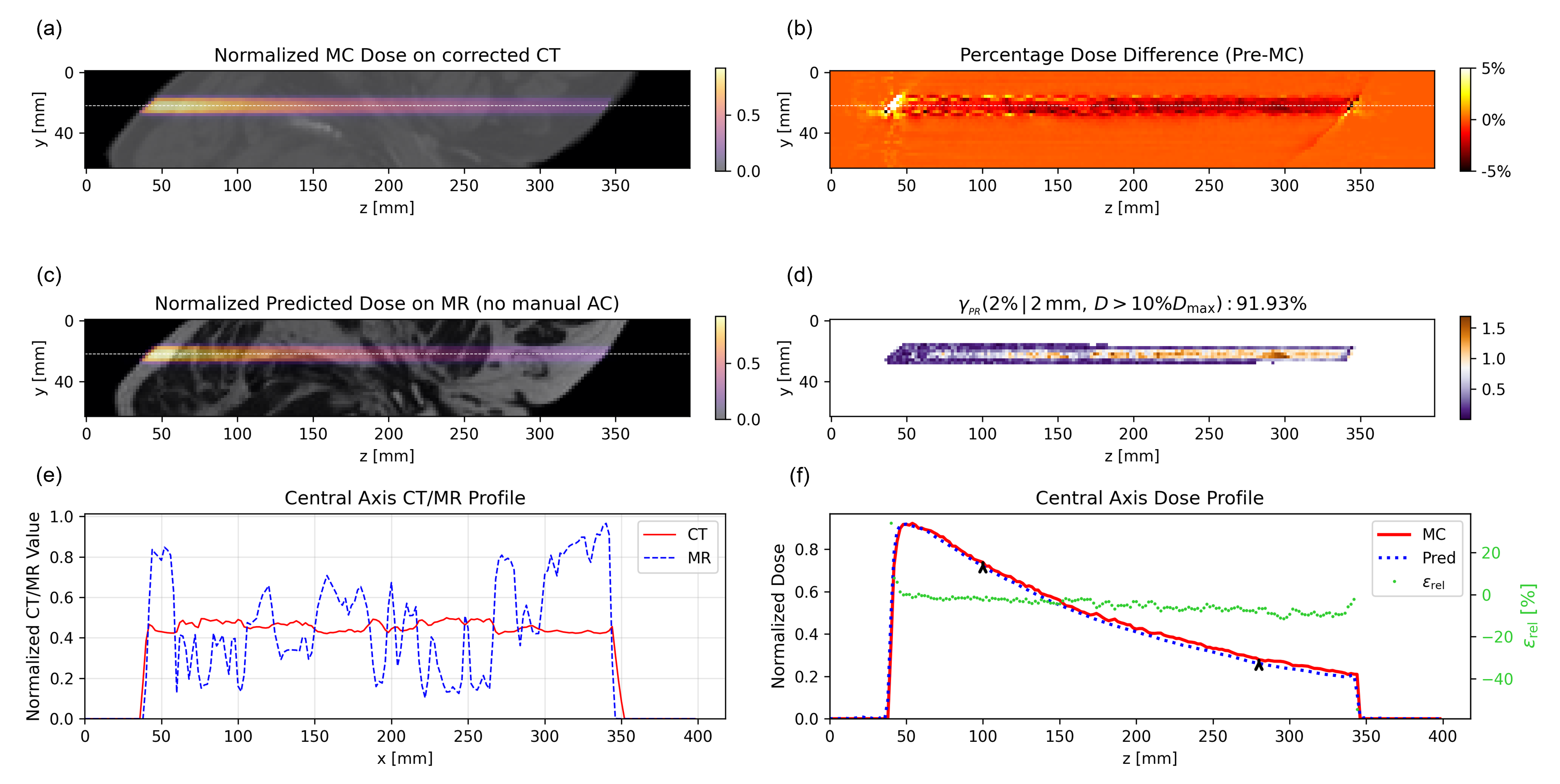


Figure S4. The worst case predicted by Model 2 (dose not through AC): *yz* slices of (a) normalized MC dose on corrected CT, (b) percentage dose difference between predicted dose and MC dose, (c) normalized predicted dose on MRI, (d) Γ (2%/2 mm) map of central dose plane, (e) the central axis value profiles of normalized CT/MR, and (f) the central axis dose profiles of normalized MC and predicted dose with the relative dose

differences. The central axis CT/MR value profiles and dose profiles are through the gray dashed line.
